# Supplementary figures and images for: Intracellular Pseudomonas aeruginosa persist and evade antibiotic treatment in a wound infection model
Source: PLoS Pathog. 2025 Feb 13;21(2):e1012922. doi: 10.1371/journal.ppat.1012922 (PMC11825101; doi:10.1371/journal.ppat.1012922)

## Slide 1
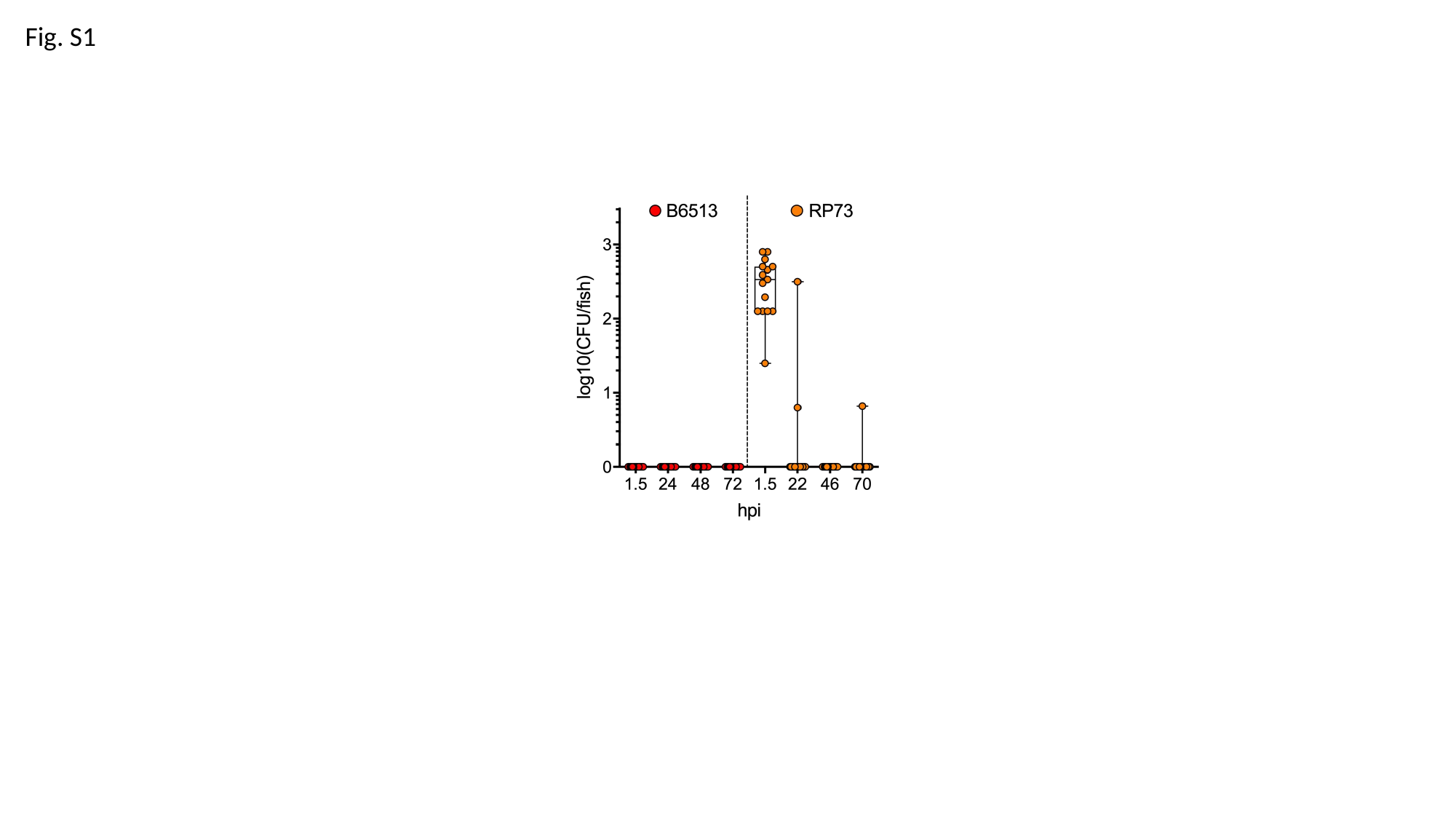

Fig. S1

Supplement: S1 Fig — Uninjured larvae were immersed with GFP+ persistent isolates B6513 and RP73, and were subsequently crushed and plated for CFU counting over approx. 72 hpi (n = 3, 15 larvae). The difference between B6513 and RP73 at time 1.5 h in uninjured embryos may reflect different ability of the strains to adhere to embryos (of note, the number of CFUs of RP73 at time 1.5 h in uninjured embryos is much lower compared to injured embryos in Fig 1C). (PPTX) [file ppat.1012922.s001.pptx]

## Slide 1
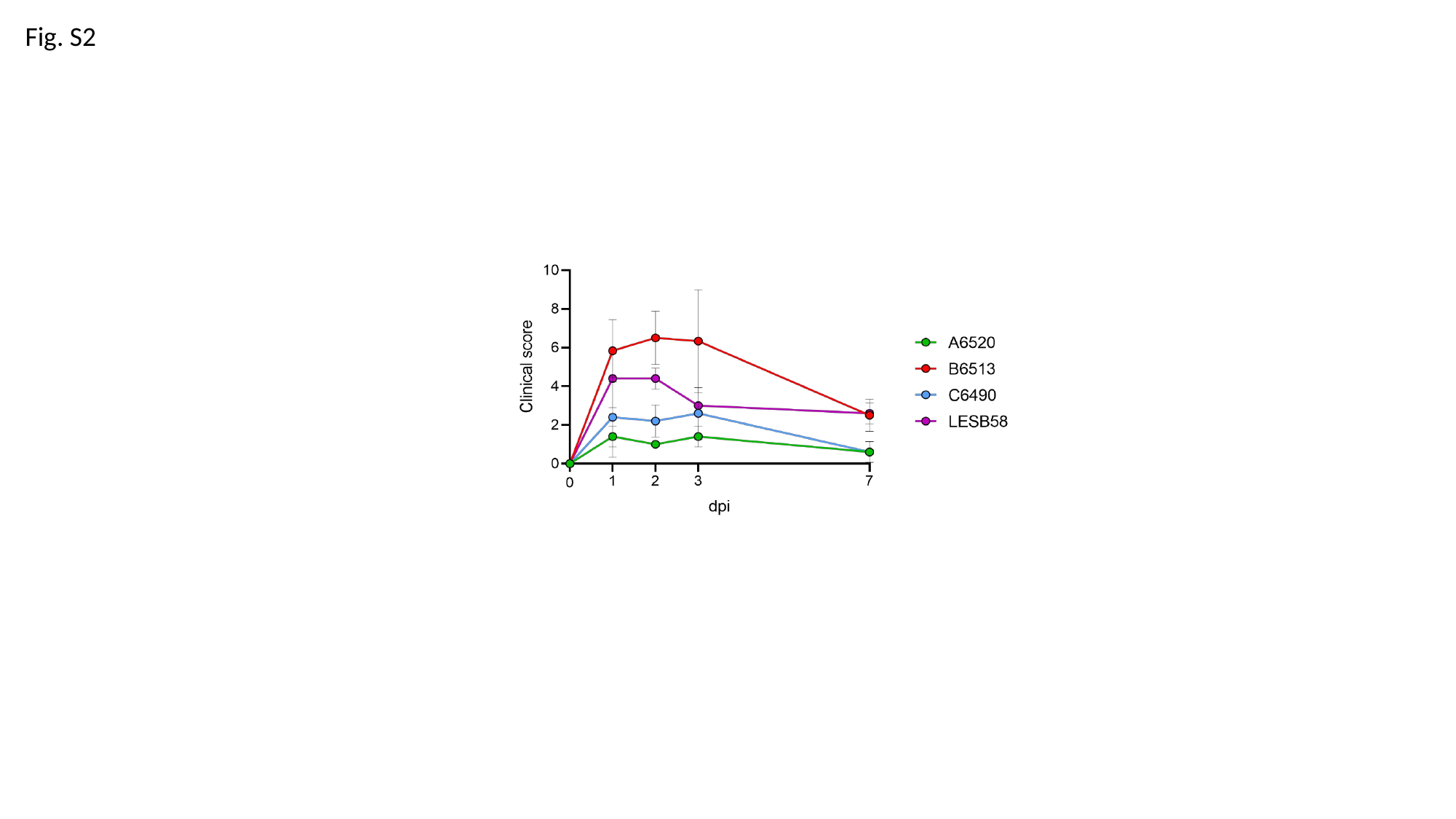

Fig. S2

Supplement: S2 Fig — Clinical welfare of animals used in the murine model of high-density cutaneous infection was monitored using a standardized scoring system for assessing disease severity. Mice were monitored daily for the first three days post-infection (dpi), then weekly thereafter. Scores were assigned for a pre-determined battery of traits, including activity, hydration, pain, injection site, etc. and then summated for each animal. Strain B6513 did cause mortality of one animal at two dpi (out of 14 infected mice), though the cause of death was not determined as total necropsy could not be performed prior to rigor mortis. Data are expressed as the mean clinical score for all animals in each treatment group +/- the standard error of the mean (SEM) (n = 5-7). (PPTX) [file ppat.1012922.s002.pptx]

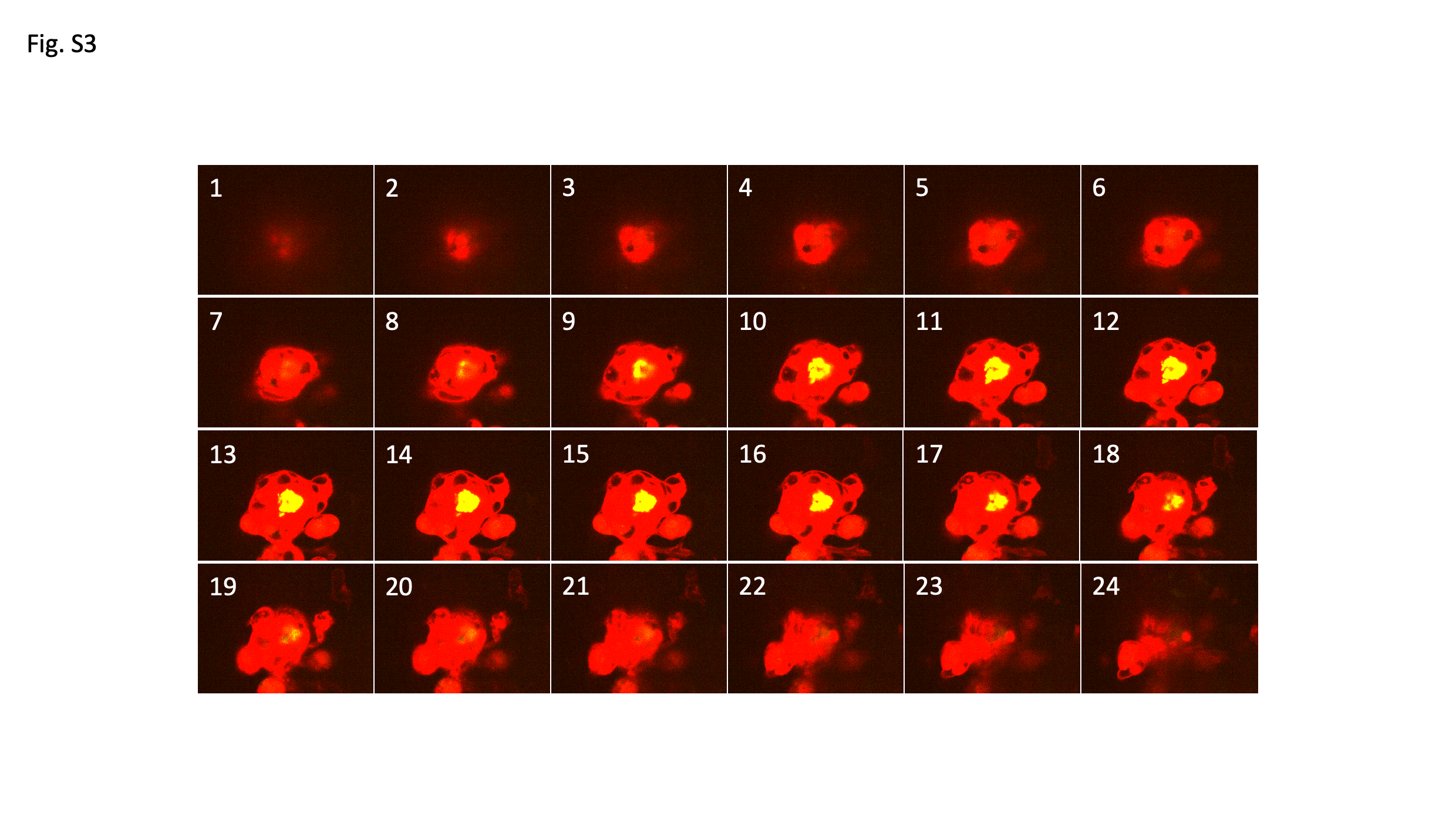

Supplement: S3 Fig — The images were used for the 3D reconstitution shown in Fig 2B, representing a bacterial foci observed at t23h. The step between each optical section is 1 μm. (TIFF) [file ppat.1012922.s003.tiff]

## Slide 1
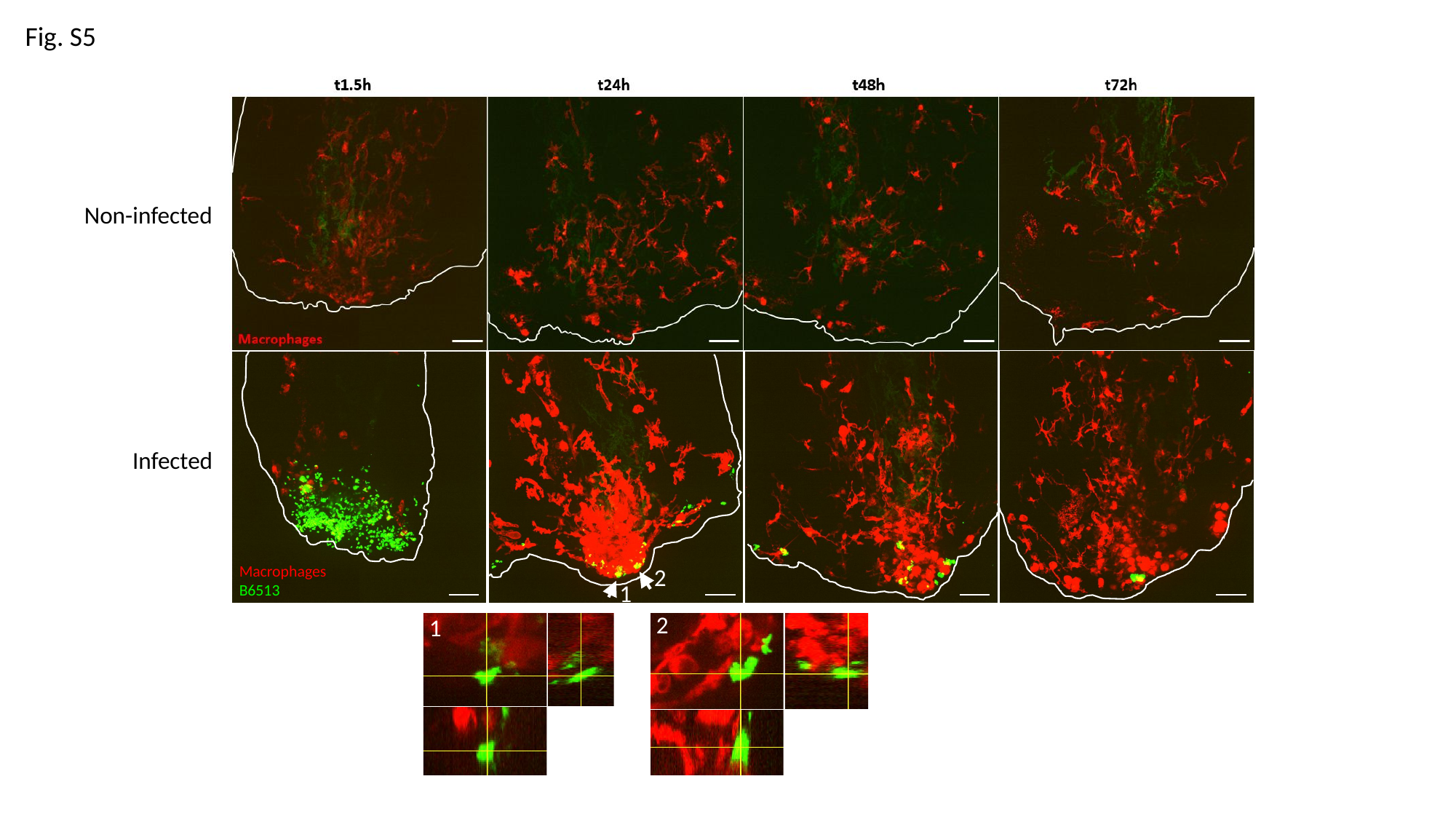

Fig. S5
Non-infected
Macrophages
B6513
Infected
2
1
2
1

Supplement: S5 Fig — Maximal projections of confocal images, showing macrophages (red) in Tg(mfap4:mCherry-F) larvae which were injured but not infected (top) or infected but without intramacrophage bacteria detected at the time of imaging (bottom). Below the images, orthogonal representations of two events (indicated by white arrows) showing that the apparent yellow color of bacterial clusters in the maximal projection does not reflect an intramacrophage localization. Scale bar: 40 µm. (PPTX) [file ppat.1012922.s005.pptx]

## Slide 1
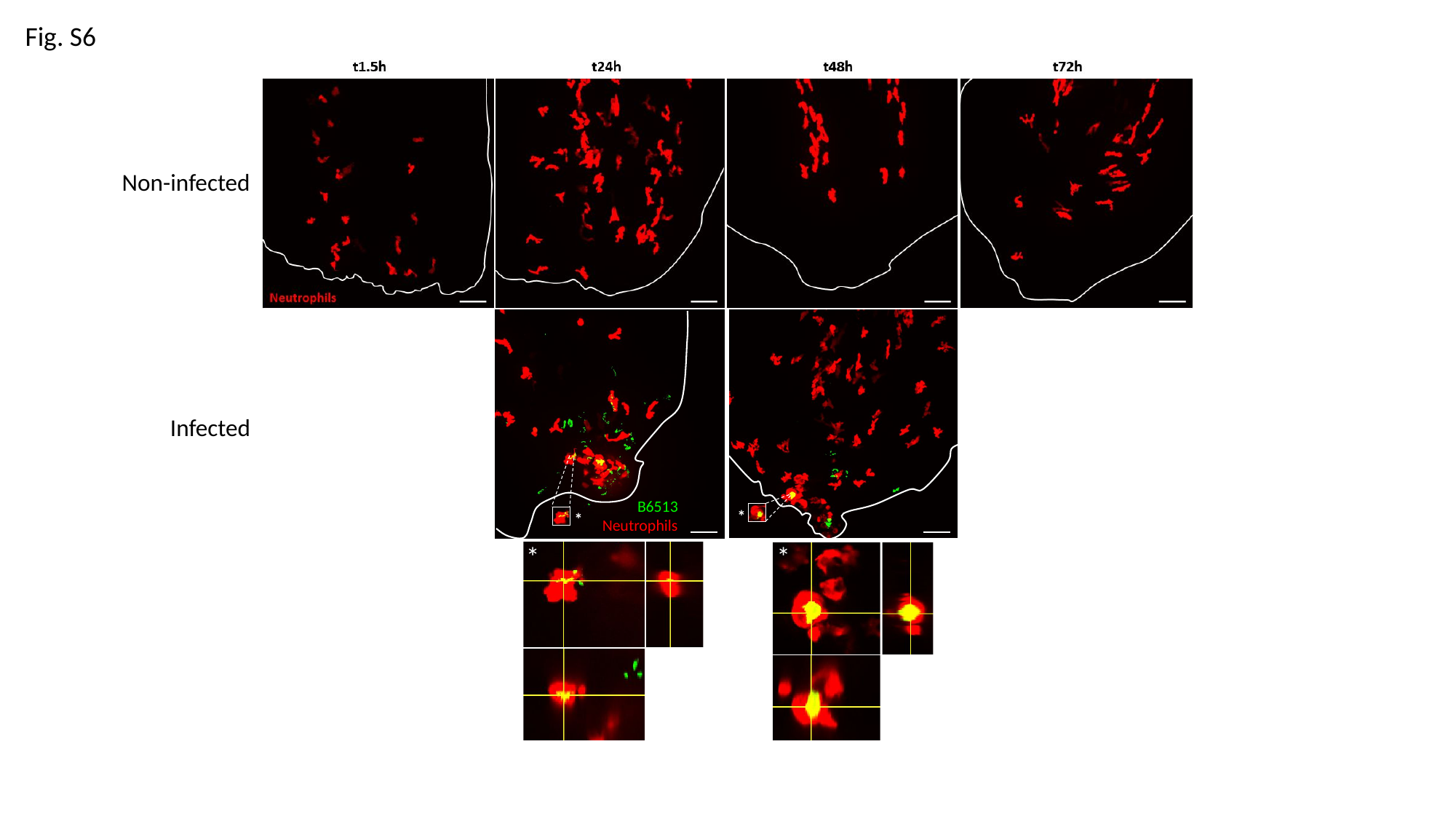

Fig. S6
Non-infected
B6513
Neutrophils
*
*
*
*
Infected

Supplement: S6 Fig — Maximal projections of confocal images, showing neutrophils (red) in Tg(LysC:dsRed) larvae which were injured but not infected (top) or infected with intraneutrophilic bacteria detected at the time of imaging (bottom). Below the images, orthogonal representations of the (*) boxed events, confirming that bacteria were intracellular. Scale bar: 40 µm. (PPTX) [file ppat.1012922.s006.pptx]

## Slide 1
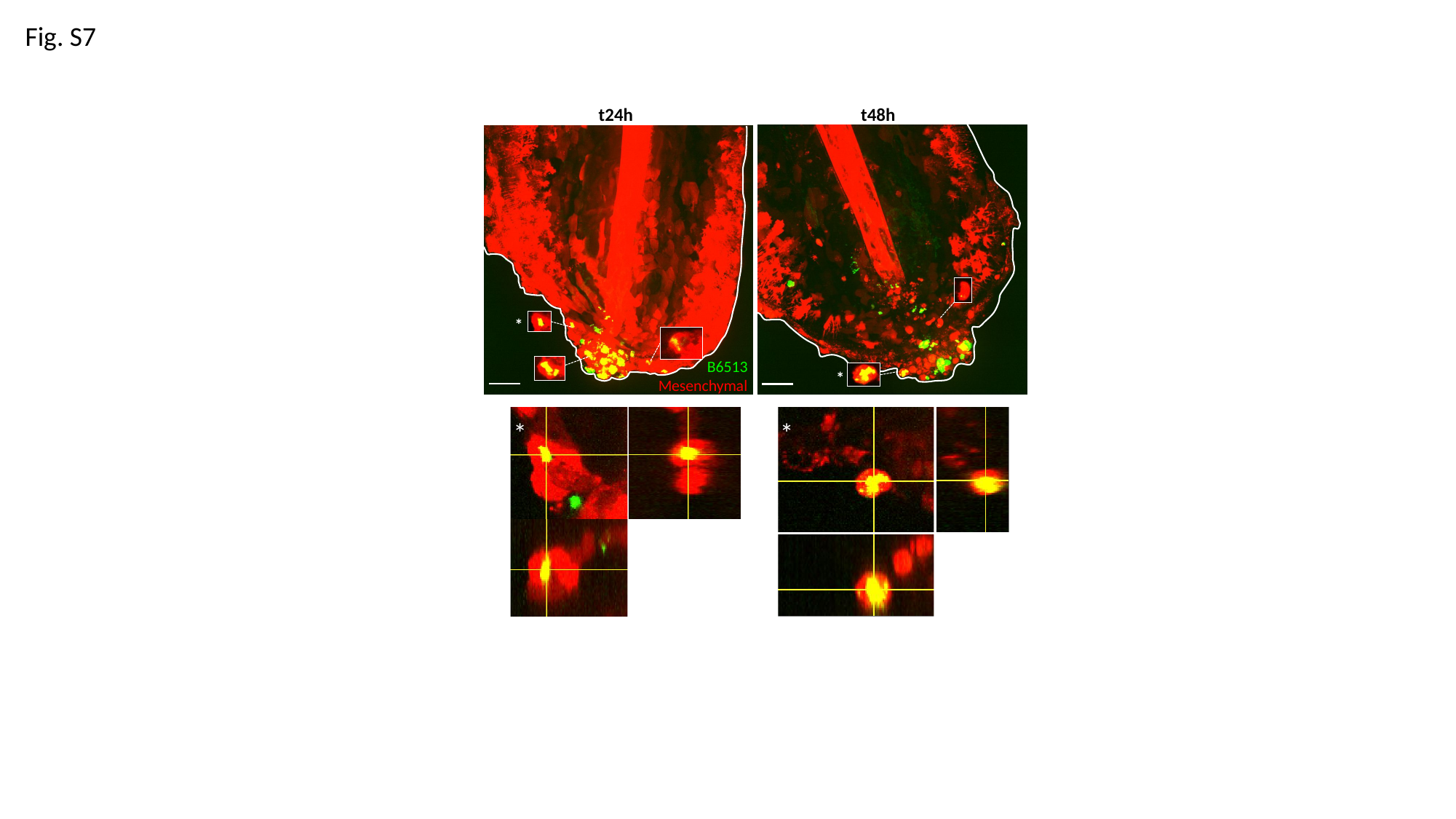

Fig. S7
t48h
*
t24h
B6513
Mesenchymal
*
*
*

Supplement: S7 Fig — Representative maximal projections of confocal images, showing interactions between bacteria (green) and mesenchymal cells (red) in Tg(rcn3:Gal4/UAS:mCherry) larvae at different time points. Boxed cells with intracellular P. aeruginosa were extracted from a single optical section. Below the images, orthogonal representations of the (*) boxed events, confirming that bacteria were intracellular. Scale bar: 40 µm. Note that pictures come from different embryos imaged for each indicated times. (PPTX) [file ppat.1012922.s007.pptx]

## Slide 1
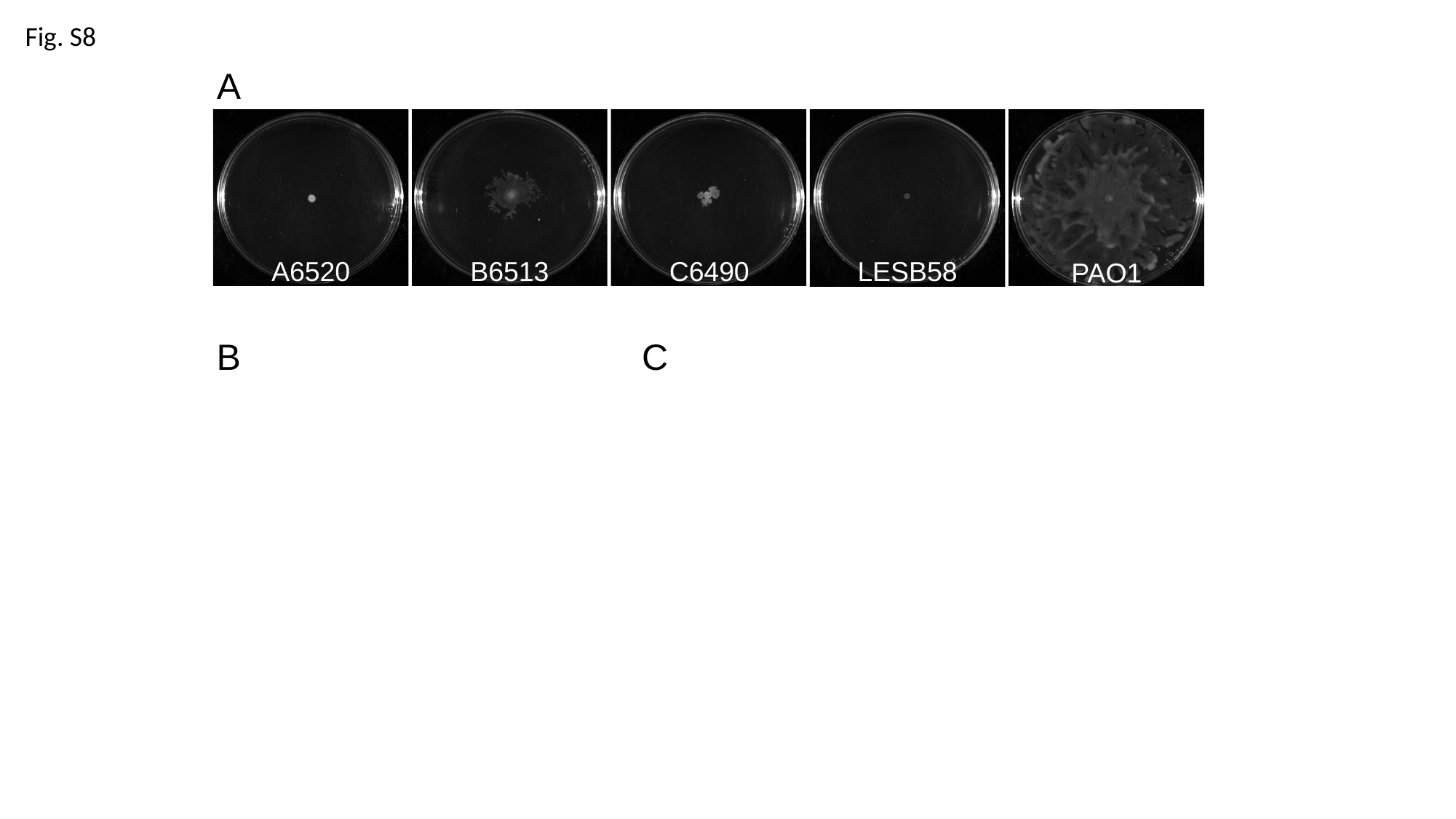

Fig. S8
A
A6520
B6513
C6490
LESB58
PAO1
B
C

Supplement: S8 Fig — (A) Representative images of swarming motility on basal medium (BM2) supplemented with 0.1% casamino acids and 0.4% agar for P. aeruginosa clinical isolates and reference strain PAO1. Images were captured with a BioRad ChemiDoc. (B) Surface area coverage of the plate was determined using FIJI (ImageJ) software. Swarming was significantly reduced in CF isolates compared to strain PAO1. The mean is displayed as a horizontal line. Significant differences (****P < 0.0001) between CF isolates and the strain PAO1 were determined by ANOVA followed by Dunnett’s post-hoc analysis. (C) Pyocyanin was extracted from the supernatants of overnight cultures and measured using a spectrophotometer (OD520nm). LESB58, which is known to overproduce pyocyanin [71], exhibited significantly increased pyocyanin production relative to PAO1 and CF clinical isolates. The mean is displayed as a horizontal line. Significant differences (*P < 0.05, ****P < 0.0001) between CF isolates and the strain PAO1 were determined by ANOVA followed by Dunnett’s post-hoc analysis. (PPTX) [file ppat.1012922.s008.pptx]

## Slide 1
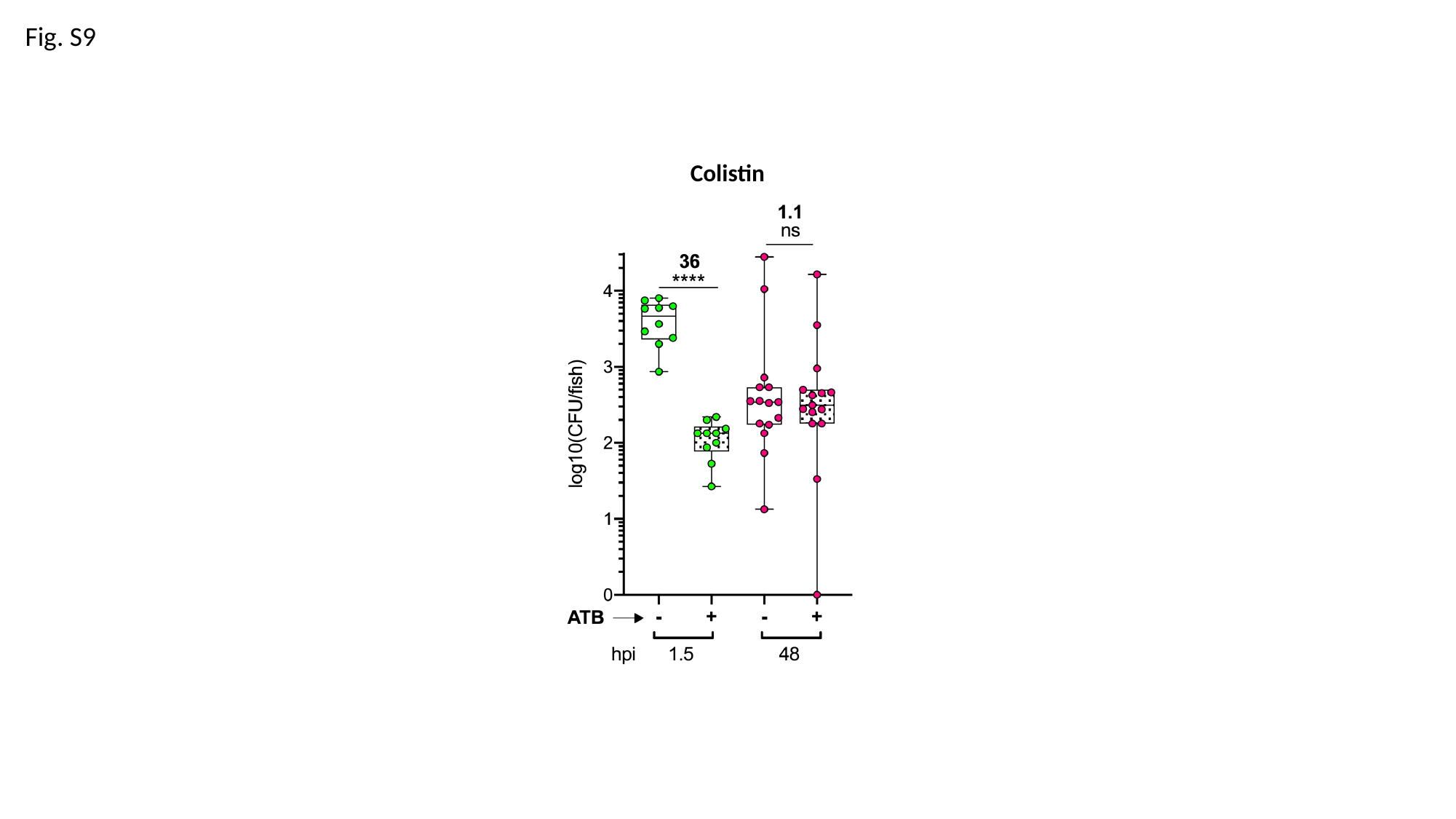

Fig. S9
Colistin

Supplement: S9 Fig — Embryos colonized for 1.5 or 48 h were subjected to colistin challenge (10 µg/ml), or incubated in water for the control condition (n = 2 to 3, 10 to 15 larvae). Following 30 min antibiotic treatment, bacterial load per embryo was determined in both groups. Mann-Whitney test: ****P < 0.0001. Ratios were calculated regarding the median of the data set. (PPTX) [file ppat.1012922.s009.pptx]

## Slide 1
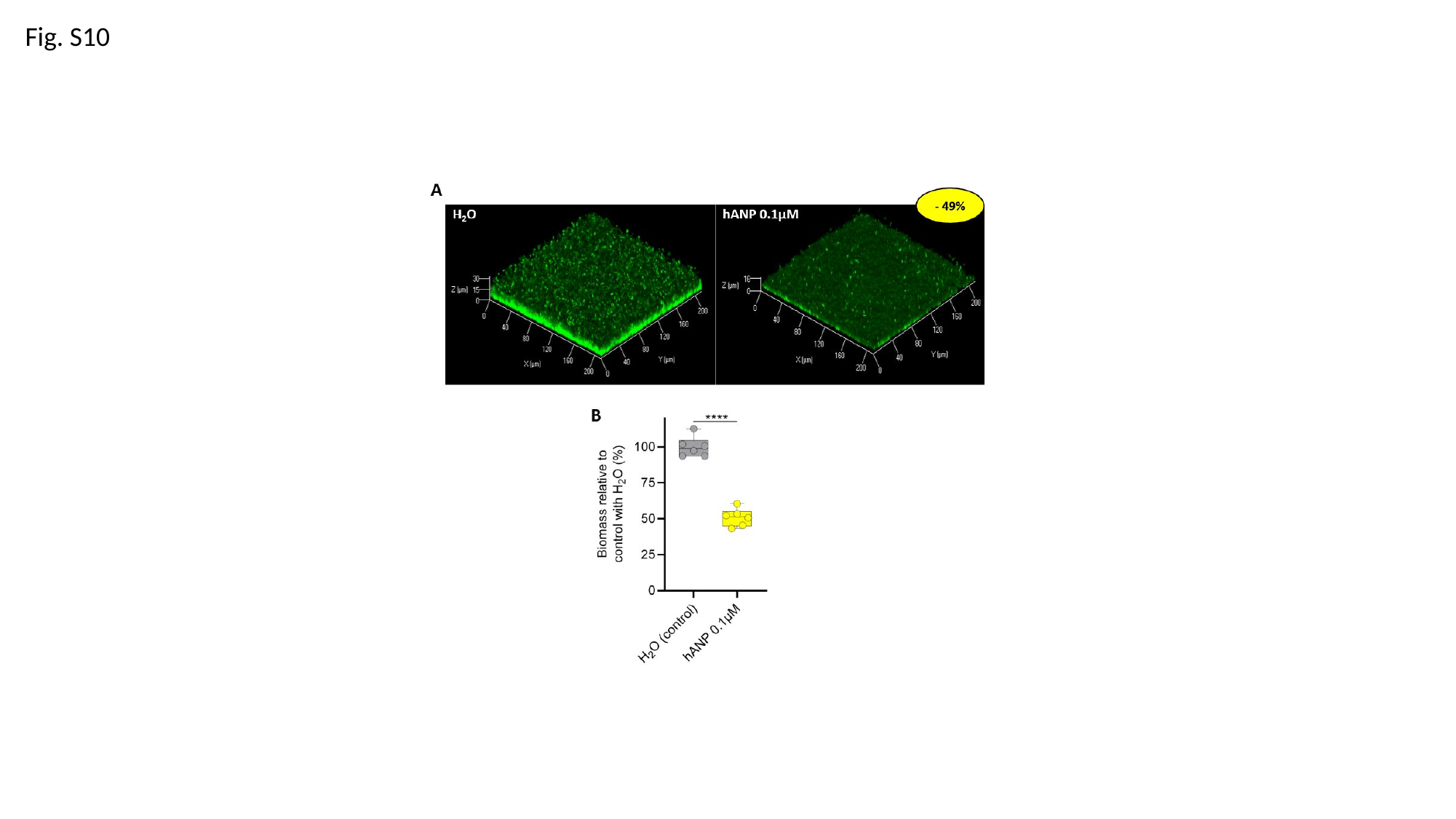

Fig. S10

Supplement: S10 Fig — (A) In vitro biofilms formed by the isolate B6513 for 24 h at 37 °C in dynamic conditions were either untreated (left) or exposed to hANP at 0.1 µM (right) for 2 h, and were imaged by confocal microscopy following SYTO9 staining of bacterial cells. (B) COMSTAT analysis of biofilms imaged in (A), six views were extracted from two independent biological experiments (n = 2). Student’s t-test: ****P < 0.0001. (PPTX) [file ppat.1012922.s010.pptx]

## Slide 1
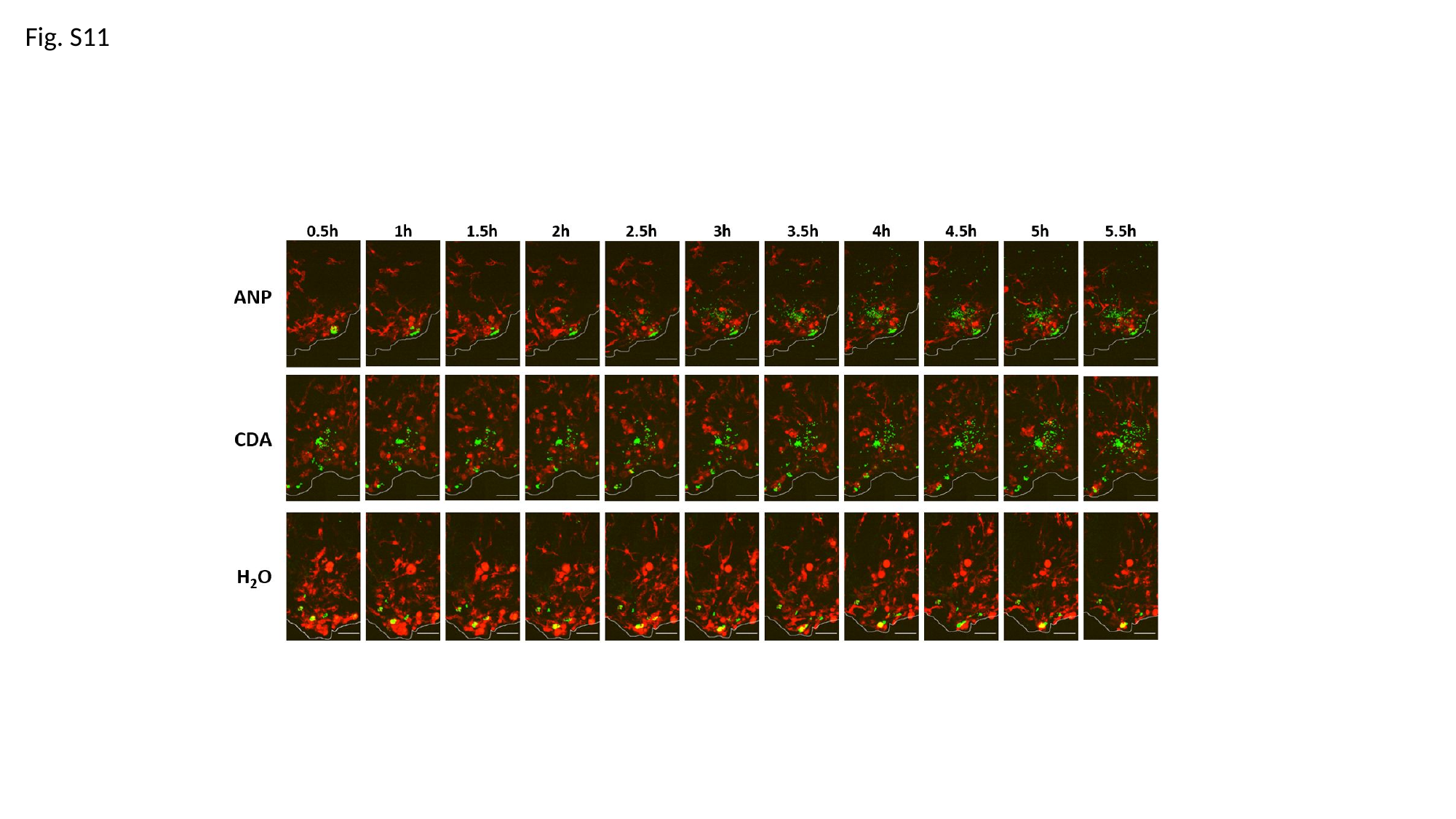

Fig. S11

Supplement: S11 Fig — Effect of hANP, CDA and H2O (control) shown in Fig 6, with a shorter time frame between images (0.5 h compared to 2.5 h). Scale bar: 40 µm. (PPTX) [file ppat.1012922.s011.pptx]
