# Supplementary material for: Intracellular Pseudomonas aeruginosa persist and evade antibiotic treatment in a wound infection model
Source: PLoS Pathog. 2025 Feb 13;21(2):e1012922. doi: 10.1371/journal.ppat.1012922 (PMC11825101; doi:10.1371/journal.ppat.1012922)
Supplement: S4 Fig — Maximal projections of confocal images, showing interactions between bacteria (green) and recruited macrophages (red) in Tg(mfap4:mCherry-F) larvae. Boxed macrophages with intracellular P. aeruginosa were extracted from a single optical sections. Below the image (infected embryos), orthogonal representation of the (*) event is shown, confirming that bacteria are inside the macrophage. (A) Left panel: embryo infected with strain RP73 at 48 hpi. Right panel: non-infected embryo. Due to a low GFP expression by strain RP73, the exposure time was increased for imaging, generating higher autofluorescence signal (mainly around the notochord). The green signal specific to bacteria was however clearly discriminated from autofluorescence based on shape, intensity, localization at the injury site and comparison with uninfected control. (B) Two larvae infected with PAO1 were imaged at 24 hpi. Events with intramacrophage bacteria are quite rare, possibly due to the lower number of persisting bacteria and/or the higher cytotoxicity of intracellular PAO1 bacteria. Scale bar: 40 µm. (PPTX) [file ppat.1012922.s004.pptx]

## Slide 1
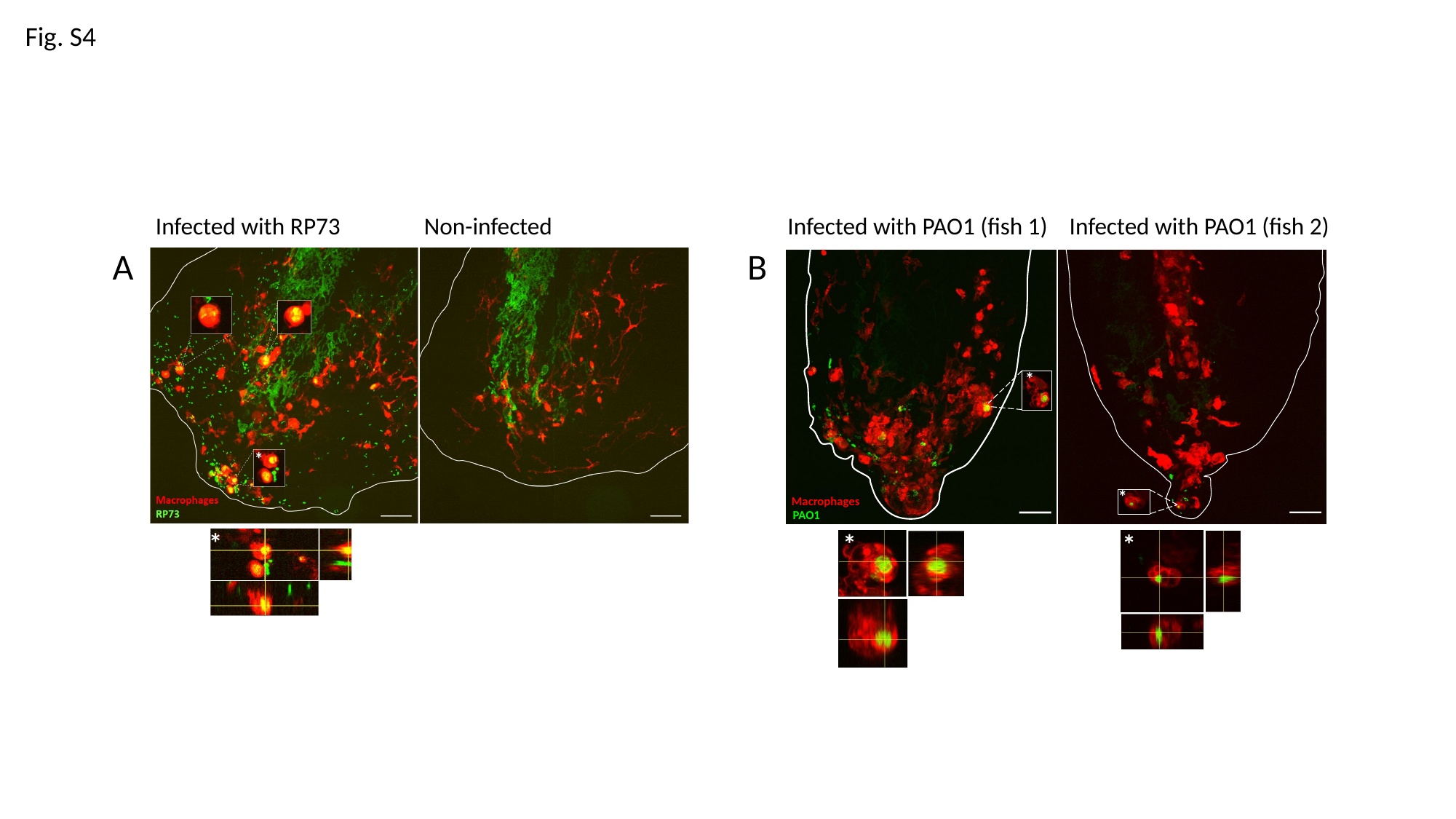

Fig. S4
Infected with RP73
Non-infected
Infected with PAO1 (fish 1)
Infected with PAO1 (fish 2)
A
*
*
B
*
*
*
Macrophages
PAO1
*
*
