# Supplementary material for: Intracellular Pseudomonas aeruginosa persist and evade antibiotic treatment in a wound infection model
Source: PLoS Pathog. 2025 Feb 13;21(2):e1012922. doi: 10.1371/journal.ppat.1012922 (PMC11825101; doi:10.1371/journal.ppat.1012922)
Supplement: S1 Table — (DOCX) [file ppat.1012922.s012.docx]

| **Glut = Glutaraldehyde; Ferro = K Ferrocyanid; UA = uranyle acetate** | | | | | | |  |  |  |  |
| --- | --- | --- | --- | --- | --- | --- | --- | --- | --- | --- |
| **Day 1** | **step number** | **time (h)** | **time (min)** | **time**  **(sec)** | **watts** | **vent**  **time** | **vac**  **time** | **set**  **vac** | **load**  **cooler T°** | **user**  **prompt** |
| Glut ON | 1 | 0 | 2 | 0 | 100 | 30 | 30 | 20 | 30 | 0 |
| Glut OFF | 2 | 0 | 2 | 0 | 0 | 30 | 30 | 20 | 30 | 0 |
| Glut ON | 3 | 0 | 2 | 0 | 100 | 30 | 30 | 20 | 30 | 0 |
| Glut OFF | 4 | 0 | 2 | 0 | 0 | 30 | 30 | 20 | 30 | 0 |
| Glut ON | 5 | 0 | 2 | 0 | 100 | 30 | 30 | 20 | 30 | 0 |
| Glut OFF | 6 | 0 | 2 | 0 | 0 | 30 | 30 | 20 | 30 | 0 |
| Glut ON | 7 | 0 | 2 | 0 | 100 | 30 | 30 | 20 | 30 | 0 |
| Buffer Rinse | 8 | 0 | 0 | 40 | 250 | 0 | 0 | 0 | 30 | 1 |
| Buffer Rinse | 9 | 0 | 0 | 40 | 250 | 0 | 0 | 0 | 30 | 1 |
| Buffer Rinse | 10 | 0 | 0 | 40 | 250 | 0 | 0 | 0 | 30 | 1 |
| OsO4 ON | 11 | 0 | 2 | 0 | 100 | 30 | 30 | 20 | 30 | 1 |
| OsO4 OFF | 12 | 0 | 2 | 0 | 0 | 30 | 30 | 20 | 30 | 0 |
| OsO4 ON | 13 | 0 | 2 | 0 | 100 | 30 | 30 | 20 | 30 | 0 |
| OsO4 OFF | 14 | 0 | 2 | 0 | 0 | 30 | 30 | 20 | 30 | 0 |
| OsO4 ON | 15 | 0 | 2 | 0 | 100 | 30 | 30 | 20 | 30 | 0 |
| OsO4 OFF | 16 | 0 | 2 | 0 | 0 | 30 | 30 | 20 | 30 | 0 |
| OsO4 ON | 17 | 0 | 2 | 0 | 100 | 30 | 30 | 20 | 30 | 0 |
| Ferro ON | 18 | 0 | 2 | 0 | 100 | 30 | 30 | 20 | 30 | 1 |
| Ferro OFF | 19 | 0 | 2 | 0 | 0 | 30 | 30 | 20 | 30 | 0 |
| Ferro ON | 20 | 0 | 2 | 0 | 100 | 30 | 30 | 20 | 30 | 0 |
| Ferro OFF | 21 | 0 | 2 | 0 | 0 | 30 | 30 | 20 | 30 | 0 |
| Ferro ON | 22 | 0 | 2 | 0 | 100 | 30 | 30 | 20 | 30 | 0 |
| Ferro OFF | 23 | 0 | 2 | 0 | 0 | 30 | 30 | 20 | 30 | 0 |
| Ferro ON | 24 | 0 | 2 | 0 | 100 | 30 | 30 | 20 | 30 | 0 |
| water Rinse | 25 | 0 | 0 | 40 | 250 | 0 | 0 | 0 | 30 | 1 |
| water Rinse | 26 | 0 | 0 | 40 | 250 | 0 | 0 | 0 | 30 | 1 |
| water Rinse | 27 | 0 | 0 | 40 | 250 | 0 | 0 | 0 | 30 | 1 |
| TCH ON | 28 | 0 | 2 | 0 | 100 | 30 | 30 | 20 | 30 | 1 |
| TCH OFF | 29 | 0 | 2 | 0 | 0 | 30 | 30 | 20 | 30 | 0 |
| TCH ON | 30 | 0 | 2 | 0 | 100 | 30 | 30 | 20 | 30 | 0 |
| TCH OFF | 31 | 0 | 2 | 0 | 0 | 30 | 30 | 20 | 30 | 0 |
| TCH ON | 32 | 0 | 2 | 0 | 100 | 0 | 0 | 20 | 30 | 0 |
| TCH OFF | 33 | 0 | 2 | 0 | 0 | 30 | 30 | 20 | 30 | 0 |
| TCH ON | 34 | 0 | 2 | 0 | 100 | 30 | 30 | 20 | 30 | 0 |
| water Rinse | 35 | 0 | 0 | 40 | 250 | 0 | 0 | 0 | 30 | 1 |
| water Rinse | 36 | 0 | 0 | 40 | 250 | 0 | 0 | 0 | 30 | 1 |
| water Rinse | 36 | 0 | 0 | 40 | 250 | 0 | 0 | 0 | 30 | 1 |
| OsO4 2 ON | 37 | 0 | 2 | 0 | 100 | 30 | 30 | 20 | 30 | 1 |
| OsO4 2 OFF | 38 | 0 | 2 | 0 | 0 | 30 | 30 | 20 | 30 | 0 |
| OsO4 2 ON | 39 | 0 | 2 | 0 | 100 | 30 | 30 | 20 | 30 | 0 |
| OsO4 2 OFF | 40 | 0 | 2 | 0 | 0 | 30 | 30 | 20 | 30 | 0 |
| OsO4 2 ON | 41 | 0 | 2 | 0 | 100 | 30 | 30 | 20 | 30 | 0 |
| OsO4 2 OFF | 42 | 0 | 2 | 0 | 0 | 30 | 30 | 20 | 30 | 0 |
| OsO4 2 ON | 43 | 0 | 2 | 0 | 100 | 30 | 30 | 20 | 30 | 0 |
| water Rinse | 44 | 0 | 0 | 40 | 250 | 0 | 0 | 0 | 30 | 1 |
| water Rinse | 45 | 0 | 0 | 40 | 250 | 0 | 0 | 0 | 30 | 1 |
| water Rinse | 46 | 0 | 0 | 40 | 250 | 0 | 0 | 0 | 30 | 1 |
| Overnight in 2% AcU 4°C then heat at 40°C (without washing) | | | | | | | | | | |
| **Day 2** | **step number** | **time (h)** | **time min** | **tim sec** | **watts** | **vent time** | **vac time** | **set vac** | **load cooler T°** | **user prompt** |
| AcU 2 ON | 1 | 0 | 2 | 0 | 100 | 30 | 30 | 20 | 30 | 1 |
| AcU 2 OFF | 2 | 0 | 2 | 0 | 0 | 30 | 30 | 20 | 30 | 0 |
| AcU 2 ON | 3 | 0 | 2 | 0 | 100 | 30 | 30 | 20 | 30 | 0 |
| AcU 2 OFF | 4 | 0 | 2 | 0 | 0 | 30 | 30 | 20 | 30 | 0 |
| AcU 2 ON | 5 | 0 | 2 | 0 | 100 | 30 | 30 | 20 | 30 | 0 |
| AcU 2 OFF | 6 | 0 | 2 | 0 | 0 | 30 | 30 | 20 | 30 | 0 |
| AcU 2 ON | 7 | 0 | 2 | 0 | 100 | 30 | 30 | 20 | 30 | 0 |
| Rinse | 8 | 0 | 0 | 40 | 250 | 0 | 0 | 0 | 30 | 1 |
| Rinse | 8 | 0 | 0 | 40 | 250 | 0 | 0 | 0 | 30 | 1 |
| Rinse | 9 | 0 | 0 | 40 | 250 | 0 | 0 | 0 | 30 | 1 |
| Lead aspartate ON | 10 | 0 | 2 | 0 | 100 | 30 | 30 | 20 | 30 | 1 |
| Lead aspartate OFF | 11 | 0 | 2 | 0 | 0 | 30 | 30 | 20 | 30 | 0 |
| Lead aspartate ON | 12 | 0 | 2 | 0 | 100 | 30 | 30 | 20 | 30 | 0 |
| Lead aspartate OFF | 13 | 0 | 2 | 0 | 0 | 30 | 30 | 20 | 30 | 0 |
| Lead aspartate ON | 14 | 0 | 2 | 0 | 100 | 30 | 30 | 20 | 30 | 0 |
| Lead aspartate OFF | 15 | 0 | 2 | 0 | 0 | 30 | 30 | 20 | 30 | 0 |
| Lead aspartate ON | 16 | 0 | 2 | 0 | 100 | 30 | 30 | 20 | 30 | 0 |
| Rinse | 17 | 0 | 0 | 40 | 250 | 0 | 0 | 0 | 30 | 1 |
| Rinse | 18 | 0 | 0 | 40 | 250 | 0 | 0 | 0 | 30 | 1 |
| Acetonitrile 50% | 19 | 0 | 0 | 40 | 250 | 0 | 0 | 0 | 30 | 1 |
| Acetonitrile 70% | 20 | 0 | 0 | 40 | 250 | 0 | 0 | 0 | 30 | 1 |
| Acetonitrile 80% | 21 | 0 | 0 | 40 | 250 | 0 | 0 | 0 | 30 | 1 |
| Acetonitrile 90% | 22 | 0 | 0 | 40 | 250 | 0 | 0 | 0 | 30 | 1 |
| Acetonitrile 100% | 23 | 0 | 0 | 40 | 250 | 0 | 0 | 0 | 30 | 1 |
| Acetonitrile 100% | 24 | 0 | 0 | 40 | 250 | 0 | 0 | 0 | 30 | 1 |
| Acetonitrile 100% | 25 | 0 | 0 | 40 | 250 | 30 | 30 | 20 | 30 | 1 |
| Resin 50 | 28 | 0 | 3 | 0 | 150 | 30 | 30 | 20 | 30 | 1 |
| Resin 75 | 29 | 0 | 3 | 0 | 150 | 30 | 30 | 20 | 30 | 1 |
| Resin 100 | 30 | 0 | 3 | 0 | 150 | 30 | 30 | 20 | 30 | 1 |
| Resin 100 | 31 | 0 | 3 | 0 | 150 | 30 | 30 | 20 | 30 | 1 |
| Resin 100 | 32 | 0 | 3 | 0 | 150 | 30 | 30 | 20 | 30 | 1 |
